# 2016 update of the PRIDE database and its related tools

**DOI:** 10.1093/nar/gkv1145

**Published:** 2015-11-02

**Authors:** Juan Antonio Vizcaíno, Attila Csordas, Noemi del-Toro, José A. Dianes, Johannes Griss, Ilias Lavidas, Gerhard Mayer, Yasset Perez-Riverol, Florian Reisinger, Tobias Ternent, Qing-Wei Xu, Rui Wang, Henning Hermjakob

**Affiliations:** 1 European Molecular Biology Laboratory, European Bioinformatics Institute (EMBL-EBI), Wellcome Trust Genome Campus, Hinxton, Cambridge, CB10 1SD, UK; 2 Division of Immunology, Allergy and Infectious Diseases, Department of Dermatology, Medical University of Vienna, Austria; 3 Medizinisches Proteom Center (MPC), Ruhr-Universität Bochum, D-44801 Bochum, Germany; 4 Department of Computer Science and Technology, Hubei University of Education, Wuhan, China; 5 National Center for Protein Sciences, Beijing, China

## Abstract

The PRoteomics IDEntifications (PRIDE) database is one of the world-leading data repositories of mass spectrometry (MS)-based proteomics data. Since the beginning of 2014, PRIDE Archive (http://www.ebi.ac.uk/pride/archive/) is the new PRIDE archival system, replacing the original PRIDE database. Here we summarize the developments in PRIDE resources and related tools since the previous update manuscript in the Database Issue in 2013. PRIDE Archive constitutes a complete redevelopment of the original PRIDE, comprising a new storage backend, data submission system and web interface, among other components. PRIDE Archive supports the most-widely used PSI (Proteomics Standards Initiative) data standard formats (mzML and mzIdentML) and implements the data requirements and guidelines of the ProteomeXchange Consortium. The wide adoption of ProteomeXchange within the community has triggered an unprecedented increase in the number of submitted data sets (around 150 data sets per month). We outline some statistics on the current PRIDE Archive data contents. We also report on the status of the PRIDE related stand-alone tools: PRIDE Inspector, PRIDE Converter 2 and the ProteomeXchange submission tool. Finally, we will give a brief update on the resources under development ‘PRIDE Cluster’ and ‘PRIDE Proteomes’, which provide a complementary view and quality-scored information of the peptide and protein identification data available in PRIDE Archive.

## INTRODUCTION

Data sharing and public availability of proteomics data requires substantial infrastructure. Several public repositories and resources have been developed in the last decade, each with different aims in mind. Among them, the PRoteomics IDEntifications (PRIDE) database was originally established at the European Bioinformatics Institute (EBI) in 2004 (1) to enable public data deposition of mass spectrometry (MS) proteomics data, providing access to the experimental data described in scientific publications. Since then, PRIDE has been evolving to fulfill the ever-growing requirements of the proteomics community (2–5). Since January 2014, PRIDE Archive (http://www.ebi.ac.uk/pride/archive/) is the new PRIDE archival resource, replacing the original PRIDE database (1).

PRIDE is one of the founding members of the ProteomeXchange (PX) Consortium (6) (http://www.proteomexchange.org) of MS proteomics resources, formally developed since 2011. It aims at standardizing data submission and dissemination of proteomics data worldwide. At present, apart from PRIDE, the resources PeptideAtlas (7), including its related resource PASSEL (PeptideAtlas SRM Experiment Library) (8), and MassIVE (Mass Spectrometry Interactive Virtual Environment, http://massive.ucsd.edu/) are the active members of the Consortium.

Within PX, PRIDE and MassIVE are focused on supporting tandem MS data (the most popular experimental approach in the field), whereas PASSEL captures Selected Reaction Monitoring (SRM) targeted proteomics data. PeptideAtlas is a secondary resource, focused on the reanalysis of tandem MS data sets using their in-house Trans Proteomic Pipeline (9). ProteomeCentral (http://proteomecentral.proteomexchange.org) is the centralized web portal for accessing all PX data sets, independently from the original resource where the data were submitted and stored.

In addition to the PX resources, there are other valuable proteomics databases and resources available, providing protein expression information derived from MS proteomics data, most notably the Global Proteome Machine Database (GPMDB) (10), ProteomicsDB (11), the Human Proteome Map (12), MaxQB (13), Chorus, PaxDb (14) and MOPED (Multi-Omics Profiling Expression Database) (15), among others. For an extensive recent review of the currently available resources, see (16). Proteomics-derived data are also made available through protein knowledge-bases such as UniProt (17) and neXtProt (for human data only) (18).

Among other data types, PRIDE Archive can store peptide and protein identifications and related quantification values, the corresponding mass spectra (both as processed peak lists and raw data), gel images, the searched sequence databases or spectral libraries, programming scripts and any other technical and/or biological metadata provided by the submitters. It is important to highlight that PRIDE Archive stores all processed results as originally analyzed by the authors. To enable data visualization and to streamline and make the data submission process easier for scientists, several open source stand-alone tools have also been made available to the proteomics community, such as PRIDE Converter (19,20), PRIDE Inspector (21) and the PX submission tool (6).

In this manuscript, we will summarize the PRIDE related developments of the last three years, since the previous *Nucleic Acids Research* (NAR) database update manuscript was published (5). We will describe the new PRIDE Archive in higher detail but will also provide information about the standalone tools and outline the ongoing quality control (QC) efforts, and finally discuss future developments.

## MAIN CHARACTERISTICS OF PRIDE ARCHIVE

**Figure 1.**
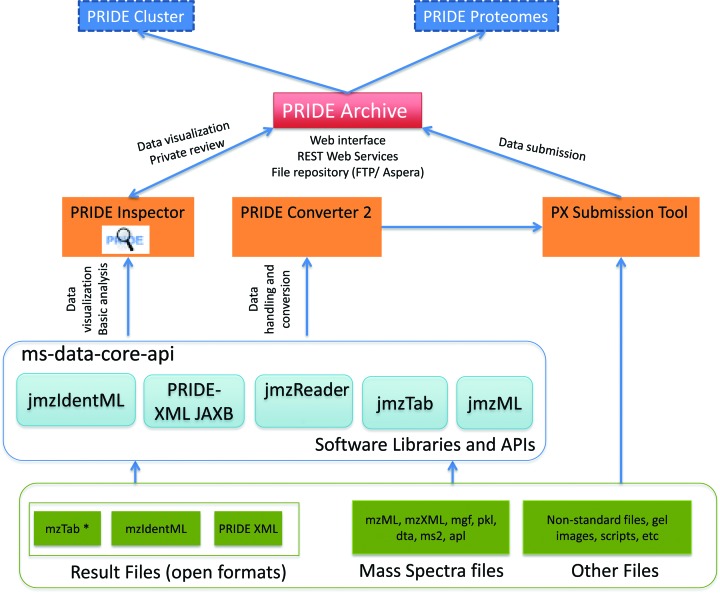
Overview of the PRIDE Archive ecosystem, including the resources (in blue, dashed lines mean resource under development), tools (in orange), software libraries and APIs (Application Programming Interfaces, in light blue) and data formats (in green). PRIDE Archive is highlighted in red.

As mentioned above, PRIDE Archive completely replaced the original PRIDE database system since the beginning of 2014. Figure 1 gives an overview of the current tools, resources and software included in the PRIDE ecosystem.

PRIDE Archive completely relies on data set submissions made by researchers. Original submitted data are not changed, but PRIDE curators can add some extra metadata annotations at the level of the data set (e.g. tagging of data sets or the published reference/s). Data sets remain private (password protected) by default and are only made publicly available after the related manuscript has been accepted, or when the PRIDE team is notified to do so by the original submitter.

### Types of data sets and support for open standard formats

PRIDE Archive is a data set or project centric resource. A data set is composed of one or more experimental assays. This approach has evolved from the original PRIDE system, where data were organised per experiment (equivalent to an ‘assay’), normally corresponding to one MS run. Following the common identifier space implemented within the PX Consortium, all PRIDE Archive data sets are now named using PXD identifiers (http://www.ebi.ac.uk/miriam/main/collections/MIR:00000513).

There are two types of data sets that can be submitted, based on the PX guidelines: ‘Complete’ and ‘Partial’. For both types, a set of common experimental metadata (agreed by all the PX partners) and raw data mass spectra are always available. The difference between both types is related to the way the processed identification results are provided.

In the case of a ‘Complete’ data set (e.g. http://www.ebi.ac.uk/pride/archive/projects/PXD000764) it is possible for PRIDE to directly connect the processed peptide/protein identification results with the submitted mass spectra. This can only be achieved if the processed results are available in a supported open data format for peptide/protein identification data. PRIDE Archive fully supports PRIDE XML (the original PRIDE data format) and the PSI (Proteomics Standards Initiative) mzIdentML data standard format for identification data (22). For the latter, the accompanying mass spectra files must also be submitted since mzIdentML does not contain actual mass spectra data. ‘Complete’ data sets can be split in assays (corresponding to each individual PRIDE XML or mzIdentML file) and as such, they can be annotated in higher detail by submitters. Assays get a secondary accession number as well (an integer number), equivalent to the identifiers given to PRIDE experiments in the original PRIDE system. All ‘Complete’ data sets also get a Digital Object Identifier (DOI) to improve the tracking of data sets and improve recognition for submitters.

In the case of ‘Partial’ data sets (e.g. http://www.ebi.ac.uk/pride/archive/projects/PXD000561), the spectra and the identification results cannot be connected in a straightforward way since the processed results are not available in an open format supported by PRIDE. Instead, the corresponding analysis software output files (in heterogeneous formats) are made available. In this case, assay numbers and DOIs are not assigned and the annotated experimental metadata is applicable to the data set as a whole. Thanks to this mechanism, data sets generated using any proteomics experimental approach (apart from data sets containing only SRM data, which are stored in the PX PASSEL resource) can now be stored in PRIDE Archive. This includes for instance top-down proteomics, data independent acquisition approaches (such as the SWATH-MS, MS^e^ and HD-MS^e^ techniques, among others) and MS imaging data sets. For the latter type of data sets, a more formal submission mechanism has been developed, involving the mandatory presence of certain MS imaging data types and files (23) (e.g. http://www.ebi.ac.uk/pride/archive/projects/PXD001283/).

Finally, the data sets available in the original PRIDE database before PX were assigned a new ‘PRD’ identifier, in addition to the original PRIDE experiment numbers. For these older data sets, raw data are not available although peak lists are available in most cases.

### Technical Implementation, including the internal file submission pipeline

All PRIDE related software is open source and written in Java. PRIDE Archive source code is available in GitHub (https://github.com/PRIDE-Archive). The software architecture of PRIDE Archive has two main components: (i) the PRIDE Archive web and web service for presenting and searching the data sets and (ii) the internal submission pipeline for handling incoming submissions and store them. Detailed technical information (including figures) is available as Supplementary File 1.

The design of the web and web service follows the ‘Layered Architecture Pattern’ (https://en.wikipedia.org/wiki/Multilayered_architecture), where each layer serves only one main responsibility and can only access the layer below (see Supplementary File 1). One of main challenges in designing such as a system is to come up with a scalable solution for storing and searching large amounts of data. We adopted a polyglot persistent model (http://martinfowler.com/bliki/PolyglotPersistence.html), where the traditional relational database and the new NoSQL (https://en.wikipedia.org/wiki/NoSQL) data stores are mixed to deliver performance. An Oracle database (http://www.oracle.com/) is used as the relational store for the biological and technical metadata, and multiple Solr stores (http://lucene.apache.org/solr/) are used for storing and enabling the search functionality for protein and peptide identifications, and spectra.

**Figure 2.**
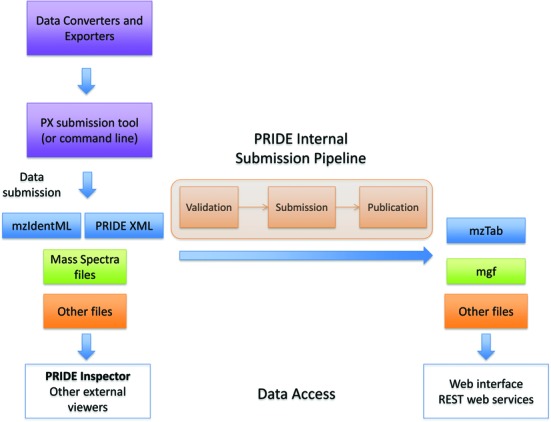
Schema representing the PRIDE submission process and internal submission pipeline, highlighting the different file formats and data types involved. The different ways to access the data are also highlighted.

The PRIDE Archive internal submission pipeline has three stages (Figure 2): (i) file validation, where schema compliance is ensured for the XML-based file formats. In addition, a report is generated that is checked by PRIDE curators to ensure that the data are correct and can be submitted (24); (ii) data submission, where the data are actually submitted to PRIDE Archive. As a result, the submitter gets the data set PXD accession number, DOI (in the case of ‘Complete’ data sets) and the reviewer credentials for accessing the data privately and (iii) data publication, to make the data publicly available once it is appropriate. In addition, some internal file conversion takes place (see below) and the information about the new data sets gets transmitted to the PX portal ProteomeCentral.

The pipeline makes use of different open source libraries that have been developed in the last years to support the PSI open standard data formats, most notably jmzIdentML (25) (for mzIdentML files), jmzML (26) (for mzML files, the PSI standard for mass spectra data (27)), jmzTab (28) (for mzTab data standard files, see below), jmzReader (29) (for a wide variety of mass spectra files) and PRIDE XML JAXB (for PRIDE XML files). In addition, a common data model is built on top of these libraries called the ms-data-core-api (30), which is essential to homogenize the process for the different data formats. It is important to highlight that these software libraries are also used by PRIDE Inspector and PRIDE Converter 2, ensuring a full compatibility between PRIDE Archive and its related standalone tools.

### How to access data

Data in PRIDE Archive can be accessed in four different ways: (i) the web interface (see details below); (ii) the recently developed REST (REpresentational State Transfer) based web services (31) (https://www.ebi.ac.uk/pride/ws/archive/), that replace the most popular functionality of the former BioMart interface, which is no longer available; (iii) a file repository, where both the FTP and Aspera file transfer protocols can be used to download the files. Aspera download functionality is accessible *via* the web interface and can be up to 1000 times faster than FTP (http://downloads.asperasoft.com/en/technology/fasp_versus_FTP_4/fasp_versus_FTP_4) and (iv) the stand-alone PRIDE Inspector tool (21) (see below).

### Data representation

All the originally submitted files, including the processed results files (PRIDE XML, mzIdentML), are not changed and always made available to download (Figure 2). Users can then use PRIDE Inspector (or other visualisation tools) to browse and visualise all the details of the experimental results. However, not all the information contained in the files is made available through the web interface and REST web services. It is however a rather large subset, which is based on the data representation available in the mzTab data standard (32). The mzTab format, the most-recently developed PSI standard, supports the reporting of identification and quantification results in a tab-delimited format.

The processed result files (mzIdentML, PRIDE XML) are converted to mzTab in the PRIDE submission pipeline whereas the different mass spectra file formats get converted to mgf (Mascot Generic File). The generated mzTab and mgf files are then used to build the backend storage of PRIDE Archive (Figure 2). Identification data coming from mzIdentML are then filtered at the web and web service level. The detailed rules about which PSMs (Peptide Spectrum Matches) are made accessible are available at http://www.ebi.ac.uk/pride/help/data-representation-pride-archive.

## PRIDE ARCHIVE DATA CONTENT STATISTICS

PRIDE Archive data content statistics are not directly comparable to the figures available in the previous NAR PRIDE update manuscripts, since the underlying data model has changed and a higher amount of data types can now be stored. Only data coming from ‘Complete’ submissions (in addition to the old PRIDE ‘PRD’ data sets) are fully incorporated into the PRIDE Archive database. At the moment of writing, these data sets (both public and private data) represent 690 M spectra, 298 M peptide identifications (corresponding to 21 M distinct peptide sequences, not considering protein modifications) and around 66 M protein identifications. Data contents corresponding to ‘Partial’ submissions are not considered here (only file sizes are recorded). In total, the size of PRIDE Archive is approximately 140 TBs.

By September 1 2015, PRIDE Archive contained 3336 data sets, of which roughly 25% are ‘Complete’ (813 data sets), 50% are ‘Partial’ (1681) and the remaining 25% (842) correspond to those stored in the original PRIDE system, before the PX data workflow was implemented. In the period March–August 2015 alone, an average of 150 data sets were submitted per month (Supplementary Figure S1), showing an unprecedented increase in data submissions. Note that these data holdings correspond to absolute figures, not distinguishing public and private data. By September 1 2015, 52% (1731) of the data sets were publicly available. The most represented species (considering both the number of data sets and the size of the stored files) are human (1342 data sets, 70 TB) and some of the main model organisms, most notably mouse (427, 28 TB), *Arabidopsis thaliana* (140, 4 TB), *Saccharomyces cerevisiae* (139, 5 TB), rat (106, 3 TB), *Escherichia coli* (67, 3 TB), cow (43, 1 TB), *Drosophila melanogaster* (32, 2 TB), chicken (25, 1 TB), rice (24, 0.2 TB), *Caenorhabditis elegans* (23 data sets, 2 TB) and soybean (23 data sets, 0.3 TB). Overall, data sets coming from more than 500 different taxonomy identifiers are stored in PRIDE Archive.

We here highlight only a very small subset of the most prominent data sets available, based on number of downloads and/or the relevance of the journal where the associated papers were published. Two of the most highly accessed data sets are those coming from the two drafts of the human proteome published in *Nature* in 2014 (11,12) (PXD000561 and PXD000865, respectively). Supplementary File 2 contains a list of the most accessed data sets in PRIDE Archive in 2015. Other prominent data sets are: a recent *C. elegans* study related to proteome remodeling and aggregation in aging (33) (PXD001364), the data sets entitled ‘Ancient proteins resolve the evolutionary history of Darwin's South American ungulates’ (34) (PXD001411), and ‘Microbial life in permafrost: predictions of microbial response to natural thaw using a multi-omics approach’ (35) (PXD001131), or the data sets ‘Progesterone receptor modulates ERα action in breast cancer’ (36) (PXD002104), and ‘Proteomics reveals dynamic assembly of repair complexes during bypass of DNA cross-links’ (PXD000490 and PXD000491) (37). For continuous information on prominent data sets, PRIDE users can follow the @pride_ebi Twitter account.

## DESCRIPTION OF THE FUNCTIONALITY OF THE PRIDE ARCHIVE WEB INTERFACE

Using the home page of PRIDE Archive (http://www.ebi.ac.uk/pride/archive/) it is possible to access all tools and resources of the PRIDE ecosystem. It is also possible to ‘Browse’ all data sets or to search for them using different criteria. Data sets can be searched and filtered based on different pieces of metadata information such as sample attributes (e.g. species, tissues, diseases, etc.), instrumentation (mass spectrometer), detected protein modifications (including the biologically relevant post-translational modifications), experiment and submission type (e.g. ‘Complete’ or ‘Partial’ submission), PubMed identifiers (for references), project tags and other experimental details that can be gathered during the data submission process. The result page of a search can be filtered further based on most of the same criteria, tailoring the final result to the needs of the users. Importantly, in addition, in the case of ‘Complete’ and older PRIDE data sets, users can search PRIDE Archive for specific protein identifiers (protein identifications), peptide amino acid sequences (peptide identifications) or even smaller sequence motifs (e.g. http://www.ebi.ac.uk/pride/archive/simpleSearch?q=*PNTIIEALR*&submit=Search).

**Figure 3.**
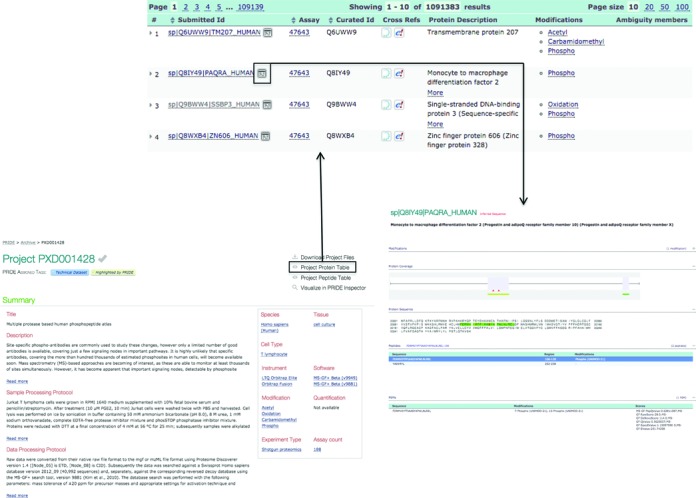
Screenshots of the PRIDE Archive web interface showing the project centric page for data set PXD001428, a section of the protein identifications table for that data set and an example of a protein identification page.

Each data set has a central project page containing a summary of the general metadata provided during the submission process (Figure 3), which includes information such as data set title, description, sample processing protocol, data processing protocol, contact (including both the submitter and the principal investigator), submission and publication dates, sample related information (species, tissue, cell type, disease, etc.), instrument, software, protein modifications or experiment type. In addition, it contains the original published reference/s, when available.

In the case of ‘Complete’ and older PRIDE data sets, at the bottom of the page, the list of assays belonging to that data set is included there, including a brief description. For each assay it is then possible to ‘Analyze’ the presence of the identified proteins in biological pathways using the new Reactome (38) Pathway Browser. Each assay has a specific assay-centric page. In this page, analogously to the project centric one, it is possible to access the specific information for each assay including more details about the instrument, search details and the experimental factor related information (if provided by the submitters), among other pieces of data.

In the case of ‘Complete’ submissions or for the relevant older PRIDE data sets, in the top right section of the data set centric page, it is possible to access four linked pages: (i) ‘Download Project files’, to download all the data set files or access directly the file repository *via* FTP or Aspera; (ii) ‘Project Protein Table’ (Figure 3), where all the identified proteins in the project are listed, including their main attributes. There, in the ‘Submitted Id’ column of the table it is possible to access the page for each protein identification; (iii) ‘Project Peptide Table’, listing all the PSMs available in the data set, including their main attributes (e.g. search engines scores, protein modifications, etc.) and (iv) ‘Visualize in PRIDE Inspector’, providing a direct link to the PRIDE Inspector tool, to visualize all the information in detail. The four same exact linked pages can also be accessed in each specific assay page in the same location. However, in the case of ‘Partial’ data sets, only the ‘Download Project Files’ link is available since, as explained above, peptide/protein identifications cannot be visualized in the web and/or in PRIDE Inspector.

The Protein Identification page (linked from the ‘Protein Table’, as explained above, Figure 3) is completely interactive. It displays the identified peptides and where possible, the protein coverage and the sequence of the identified protein. All the PSMs sharing the same sequence (combination of amino acids and modifications) are grouped in the ‘Sequence’ panel, but split in the ‘PSMs’ panel below. It is important to highlight that often, it may not be possible to provide the corresponding exact protein sequence for the reported identified peptide sequences if the protein sequence was not included in the originally submitted files (39). In these cases, PRIDE tries to infer the sequence (something that is highlighted in the page), accessing the most updated version of the particular protein sequence in external resources. At present, the supported reference systems for protein identifications are UniProt and Ensembl. Protein identifiers are mapped using the UniProt ID mapping service (http://www.uniprot.org/mapping/).

## PRIDE DATA SUBMISSION PROCESS AND RELATED TOOLS

The data submission process to PX *via* PRIDE (both for ‘Complete’ and ‘Partial’ submissions) is described in detail here (40). In addition, a web tutorial is available here (http://www.proteomexchange.org/sites/proteomexchange.org/files/documents/px_submission_tutorial.pdf) and in the EBI e-learning platform (http://www.ebi.ac.uk/training/online/course/proteomexchange-submissions-pride). Users can however use the PRIDE related stand-alone tools to streamline the process.

### File conversion tools

As mentioned before, to perform a ‘Complete’ submission, submitters must provide the identification results in either the PRIDE XML and/or mzidentML data format. At present, PRIDE provides the PRIDE Converter 2 (20) (available at https://github.com/PRIDE-Toolsuite/pride-converter-2) as a conversion tool of different proteomics output formats into the PRIDE XML format. Details of the supported formats can also be found here (20). In addition, there are also other external tools that can export PRIDE XML files such as the ProteinLynx Global Server (PLGS, Waters Corporation) (40) and EasyProt (41). However, we encourage submitters to use the mzIdentML format (version 1.1) by default for performing the data submissions. The main reason is that mzIdentML (together with the accompanying mass spectra files) enables a richer and more accurate data representation than PRIDE XML.

Fortunately, in the last two years, it is becoming increasingly common that popular proteomics tools and analysis pipelines export mzIdentML natively. This includes, among others, MS-GF+ (42), Mascot (*Matrix Science*, from version 2.4), ProteinPilot (*AB SCIEX*), PEAKS (*Bioinformatics Solutions Inc*), Scaffold (*Proteome Software*), MyriMatch (43) and PeptideShaker (44) and recently X!Tandem (from the PILEDRIVER version) (45). For a comprehensive list of the software exporting mzIdentML, see Supplementary Table S1.

This means that, generally, conversion of files is becoming less of a burden for submitters, streamlining the whole submission process. However, there are still some highly-used tools without mzIdentML export capability, e.g. the popular Proteome Discoverer (*Thermo Scientific*) and MaxQuant (46). In these cases, while waiting for this functionality to be available, a ‘Partial’ submission is the alternative way to make the data sets available.

### PRIDE Inspector

The PRIDE Inspector stand-alone tool was originally developed in 2010–2011, enabling researchers to visualize and perform an initial quality assessment of the data before and after submission to PRIDE Archive (21). The original version supported the PRIDE XML and mzML formats, in addition to providing access to the public data in PRIDE Archive. The need to support the new PSI data standards such as mzIdentML and mzTab, and the feedback received from users resulted in the development of a new version of the tool with extended visualisation capabilities.

The new PRIDE Inspector (available at https://github.com/PRIDE-Toolsuite/pride-inspector) now extends support to cover several different experimental output files, ranging from mass spectra (mzML, mzXML and the most popular peak lists formats such as mgf, dta, ms2, pkl and apl), peptide and protein identification results (mzIdentML, PRIDE XML, mzTab), to quantification data (mzTab, PRIDE XML). In addition to supporting new data formats, novel features and capabilities are also available in the new version of the tool. While we have significantly enhanced PRIDE Inspector features, we unfortunately had to turn of the direct Java Web Start functionality in the PRIDE Archive web interface, in common with many other tools using this platform, due to security concerns.

### The PX submission tool

The PX submission tool (available at http://www.proteomexchange.org/submission-proteomexchange-pride) can be used to perform the data submission to PRIDE Archive. Using a graphical user interface the tool can: (i) select all the files before submission; (ii) group them (e.g. the corresponding raw mass spectra and processed results files); (iii) add experimental metadata annotation and (iv) upload all the files to the EBI.

The tool has been in maintenance mode since January 2014, and only a few refinements had to be implemented since. The biggest improvement, implemented in summer 2014, was the addition of the much faster Aspera file transfer protocol as the default way to transfer the files. This makes data submissions much more straightforward. However, the tool can automatically switch to FTP if Aspera cannot be enabled due to the local computer infrastructure settings. It must also be highlighted that it is possible to perform a batch submission of files using a command line functionality (for details see (40)).

## QUALITY CONTROL IN PRIDE: PRIDE CLUSTER AND PRIDE PROTEOMES

In the previous NAR manuscript update (5), we reported that we were working in the development of ‘PRIDE-Q’, a resource that would show the high-quality identification data coming from PRIDE. The name of this resource was later changed to ‘PRIDE Proteomes’. Here, we are just giving a brief update about this new PRIDE resource (in beta, http://wwwdev.ebi.ac.uk/pride/proteomes/). PRIDE Proteomes provides a quality-filtered, across-dataset view on the identification data available in PRIDE Archive.

The PSMs reported in PRIDE Archive are first quality-filtered using a spectrum clustering approach: all the identified spectra coming from the public experiments in PRIDE Archive are clustered using a refined version of the ‘PRIDE Cluster’ algorithm (47). The results of the process are made available through the peptide centric PRIDE Cluster resource (http://www.ebi.ac.uk/pride/cluster/). Good-quality spectrum clusters (for details on the criteria see (47)) are then used as evidence for peptide sequences, which are used to populate PRIDE Proteomes, which is a protein-centric resource. The peptide sequences (coming from PRIDE Cluster) are mapped to protein sequences coming from the corresponding species-specific UniProt ‘complete proteomes’ database. In PRIDE Proteomes, the information is split in different species, human being the first one that will be formally released. At present we are still working on refining the system. The new resource will be formally released as soon as possible, and will provide the researchers a complementary way to access PRIDE data.

## PRIDE DATA REUSE

Approaches based on the reanalysis of public data sets and/or promoting new uses of the stored data are becoming more popular (for a recent review see (48)). For instance, in 2014 alone, more than 160 TBs of data were downloaded from PRIDE Archive.

First of all, data sets can be reanalyzed using different analysis pipelines and/or sequence databases to discover, confirm or highlight new biological evidences. Resources such as GPMDB and PeptideAtlas have been reanalyzing PRIDE data sets for many years already, for instance in the context of the Human Proteome Project (49). Furthermore, targeted reanalysis by individual groups is now starting to be common. Remarkably, one of the published drafts of the human proteome (11) includes a big proportion of reanalyzed public data sets (≈40% of all the MS runs included), many of them coming from PRIDE Archive. Importantly, the fact that both drafts of the human proteome have been made available in PRIDE Archive (see Section 3) has enabled the community to start a lively discussion about the reliability of the results (50).

In addition, data from PRIDE Archive are then being reused in different ways: (i) the design of SRM/MRM (Multiple Reaction Monitoring) transitions, for instance by the MRMAID resource (51) and more recently in the PeptidePicker pipeline (52,53); (ii) the building of spectral libraries: PRIDE spectral libraries for several species are available *via* PRIDE Cluster at http://www.ebi.ac.uk/pride/cluster/#/libraries; (iii) to enrich sequence annotations by protein knowledge-bases such as UniProt (17), especially at the level of the evidence that supports protein existence and (iv) in *meta*-analysis studies, analyzing data in a combined way coming from a large number of data sets. In recent years, data from PRIDE have been used in several studies of this type, among many others to predict fragmentation patterns (54) or to study the performance of trypsin (55).

Finally, the already mentioned PeptideShaker standalone tool (44) has been recently developed enabling the post-processing and visualization of proteomics experiments. In this reanalysis context, it makes straightforward the direct reprocessing of public PRIDE Archive data sets by using the ‘PRIDE Reshake’ functionality.

Moving outside the proteomics field, reprocessing and/or reuse of similar data sets generated by different 'omics' approaches or by the same samples in ‘multi-omics’ studies is now starting to happen. We here highlight a recent paper on psoriasis (56) where public transcriptomics data and proteomics data from PRIDE Archive was integrated with the data originally generated in the study.

## DISCUSSION AND FUTURE PLANS

In the last three years, in the framework of the PX Consortium, PRIDE Archive has established itself as the world-leading resource for the storage of MS proteomics experiments. In this context, in the near future, we plan to support mzTab as a submission format as well, by extending our internal submission pipeline and the PX submission tool. We see mzTab as the ideal way to support quantitative results. In this context, it is important to highlight that PRIDE Inspector already supports the visualization of both identification and quantification mzTab files. It would be possible to support mzQuantML files (the PSI data standard for quantification data) (57) by converting those files to mzTab as well. As usual, the original file would be made available to download and the information in higher level of detail could always be visualized using other standalone tools.

In the context of the QC effortspride, we plan to release the PRIDE Proteomes resource as soon as possible and we will work toward the tight integration between the three resources: PRIDE Archive (original data), PRIDE Cluster (peptide centric, based on spectrum clusters) and PRIDE Proteomes (protein centric). In addition, we envision the use of the recently developed qcML format (58) to provide QC information about the data sets available in PRIDE Archive.

In addition to the work that has been done for many years by resources such as PeptideAtlas and GPMDB, data reprocessing efforts are flourishing in the field (11,59). In this context, on the one hand, we plan to increase the level of annotations for samples to facilitate data discovery for reprocessing purposes (60). On the other hand, we plan to integrate reprocessed data sets in the context of the originally submitted ones in PRIDE Archive, enabling a comparison between the reported identification results.

Finally, we want to highlight that up-to date documentation is available at the PRIDE Archive website. For instance, an FAQ (Frequently Asked Questions) section (including ‘Troubleshooting’) is available at http://www.ebi.ac.uk/pride/help/archive/faq. As mentioned before, we invite interested parties in PRIDE related developments to follow the PRIDE Twitter account (@pride_ebi). For regular announcements of all the new publicly available data sets, users can follow the PX Twitter account (@proteomexchange) or subscribe to a dedicated RSS feed (https://groups.google.com/forum/feed/proteomexchange/msgs/rss_v2_0.xml).
