# 2016 update of the PRIDE database and its related tools

**DOI:** 10.1093/nar/gkw880

**Published:** 2016-09-28

**Authors:** Juan Antonio Vizcaíno, Attila Csordas, Noemi del-Toro, José A. Dianes, Johannes Griss, Ilias Lavidas, Gerhard Mayer, Yasset Perez-Riverol, Florian Reisinger, Tobias Ternent, Qing-Wei Xu, Rui Wang, Henning Hermjakob

**Affiliations:** 1 European Molecular Biology Laboratory, European Bioinformatics Institute (EMBL-EBI), Wellcome Trust Genome Campus, Hinxton, Cambridge, CB10 1SD, UK; 2 Division of Immunology, Allergy and Infectious Diseases, Department of Dermatology, Medical University of Vienna, Austria; 3 Medizinisches Proteom Center (MPC), Ruhr-Universität Bochum, D-44801 Bochum, Germany; 4 Department of Computer Science and Technology, Hubei University of Education, Wuhan, China; 5 National Center for Protein Sciences, Beijing, China

*Nucl. Acids Res*. (04 January 2016) 44 (D1): D447–D456. doi: 10.1093/nar/gkv1145.

The authors wish to correct the affiliation of one of the authors. Gerhard Mayer's only affiliation should be ^3^Medizinisches Proteom Center (MPC), Ruhr-Universität Bochum, D-44801 Bochum, Germany.

He does NOT have a second affiliation at: ^1^European Molecular Biology Laboratory, European Bioinformatics Institute (EMBL-EBI), Wellcome Trust Genome Campus, Hinxton, Cambridge, CB10 1SD, UK.

The authors apologise to the readers for this error.

